# Biomarkers for cancer cachexia: where do we stand?

**DOI:** 10.1002/jcsm.12641

**Published:** 2020-11-30

**Authors:** Sandra Palus, Jochen Springer

**Affiliations:** ^1^ Berlin Institute of Health Center for Regenerative Therapies (BCRT) Charité Universitätsmedizin Berlin Berlin Germany; ^2^ German Centre for Cardiovascular Research (DZHK) partner site Berlin Charité Universitätsmedizin Berlin Berlin Germany

**Keywords:** Cancer cachexia, Lipids, Sphingolipids, Biomarker

Cancer cachexia has been recognized as a major, life‐limiting complication in the treatment of cancer patients and is composed of distinctive stages: pre‐cachexia, cachexia, and refractory cachexia.^1^ An early diagnosis of cachexia, possibly in the pre‐cachectic state, in cancer patients based on biomarkers would be extremely beneficial in the struggle to fight the multifactorial syndrome. Potential biomarker should not only be predictive but should also allow to monitor the progression of cachexia and the effects of putative therapies. However, while there are a number of biomarker candidates (for comprehensive overview, see Loumaye and Thissen^2^) such as TGF‐β,^3^ activin A,^4^ myostatin,^5^, ^6^ systemic inflammation,^7^ pro‐inflammatory cytokines and chemokines,^8^, ^9^, ^10^ micro RNAs,^11^, ^12^, ^13^, ^14^, ^15^, ^16^, ^17^, ^18^ and protein degradation products,^19^, ^20^, ^21^ none have been clinically established, except for the hallmark symptom weight loss in combination with additional factors such as muscle mass and strength.^22^ In addition, markers of fat loss such as leptin,^23^, ^24^ free fatty acids,^25^, ^26^ glycerol,^27^ and zinc‐α2‐glycoprotein^28^ may be of clinical interest.

In the current issue of the *Journal of Cachexia, Sarcopenia and Muscle*, Morigny *et al*. have addressed the alterations in bioactive lipids associated with cancer cachexia in pre‐clinical mouse models (Colon‐26, LLC, and APC^Min/+^) and complemented the results with the analysis of cancer patients with or without cachexia. The lipidome analysis performed by the authors included 1100 lipid species, and the results show that several bioactive lipids were regulated in cachexia. Sphingolipids (for comprehensive description of the sphingolipid metabolism, see Gault *et al*.^29^) were associated with the severity of cachexia. Most importantly, the regulation of sphingomyelin, ceramide, and hexosylceramides (16:0 and 24:1) allowed an early detection of cachexia, thus making them interesting biomarker candidates that should be validated retrospectively in material from completed clinical trials as well as in prospective clinical studies in a timely manner. Preferably, a complete analysis of the plasma lipidome should be performed, which leads to the problems of cost and availability of the (FIA)‐mass spectrometry/mass spectrometry (MS/MS) platform that was utilized by the authors of this paper. However, the lack of established biomarkers that can detect pre‐cachexia or early cachexia before the weight loss and/or low skeletal muscle criteria are met makes it imperative to invest heavily into the development of biomarkers like those that are discussed in the paper of Morigny *et al*.^30^

## Conflict of interest

The authors have no conflict of interest.
